# Frictions of implementing EU humanitarian aid in Greece (2016–2019)—the Emergency Support Instrument and its practical ramifications

**DOI:** 10.1186/s41018-021-00095-7

**Published:** 2021-05-17

**Authors:** Cordula Dittmer, Daniel F. Lorenz

**Affiliations:** Disaster Research Unit (DRU), Freie Universität Berlin, Carl-Heinrich-Becker-Weg 6-10, 12165 Berlin, Germany

**Keywords:** Greece, Refugee crisis 2015/2016, Emergency Support Instrument, DG ECHO, Humanitarian aid, Frictions, EU

## Abstract

With the closure of the border with then-Macedonia in early 2016, it was foreseeable that Greece would become the “last station” for a large number of refugees. Flanked by the agreement between Turkey and the European Union of March 2016, Greece underwent a profound transformation from a transit country to a recipient country. Through a new regulation, the Emergency Support Instrument, initially activated by the European Commission 2016–2019, international humanitarian aid operations were supported for the first time in the EU. The article analyzes the resulting frictions on the basis of empirical field research and a broad literature review. While frictions similar to those in other non-European humanitarian operations exist, specific peculiarities due to the operation taking place in an austerity-ridden member state of the EU must also be noted.

## Introduction

With the closure of the border with then-Macedonia in early 2016, it was foreseeable that Greece would become the “last station” for a large number of refugees. Flanked by the agreement between Turkey and the European Union (EU) in March 2016 (European Council 2016), Greece underwent a profound transformation from a transit country to a recipient country. This transformation was accompanied by the entry of the Directorate-General for European Civil Protection and Humanitarian Aid Operations (DG ECHO), a major donor of international humanitarian aid, and the related UN organizations, Red Cross and Red Crescent Societies and international nongovernmental organizations (INGOs). Through a new regulation, the Emergency Support Instrument (ESI), the EU contracted €644.5 million from 2016 to 2019 for projects in all administrative regions in Greece to support the Greek government or UN organizations and implementing INGOs with the provision for refugees (DG ECHO 2018; European Commission 2019). Although the DG ECHO itself considered the mission a success (DG ECHO 2018), it has been widely criticized by public media and humanitarian actors. First, the mission is seen as one of the most expensive and ineffective international humanitarian operations ever. Secondly, international humanitarian aid actors claimed that humanitarian funds should be spent on more severe humanitarian crises rather than to support an EU member state with a social and welfare system. Third, it was criticized that humanitarian aid was implemented in an EU context without considering that it was originally established for non-European regions (DeLargy 2016; Howden and Fotiadis 2017; van Pottelbergh et al. 2019). This paper analyzes frictions that arose from the complex interplay of the new instrument, the international humanitarian actors, Greek authorities and the overall situation in Greece. Most significant for the context is that Greece, at that time, had to overcome a financial and debt crisis that has been ongoing since 2007, as well as corresponding austerity measures that affected the social and welfare sector in particular.

In the first part of the paper, we will give a brief overview of the historic socioeconomic and political situation in Greece relating to the financial crisis and austerity measures as well as the refugee situation since 2013. After that, we develop our conceptual and theoretical framework and present our methodological approach. In addition to empirical fieldwork in 2017 and 2019, we include the findings of research on humanitarianism, which has thus far dealt exclusively with non-EU contexts due to a lack of empirical cases. We combine those findings with the frictions approach (Björkdahl et al. 2016; Björkdahl and Höglund 2013; Tsing 2005). That approach allows for an analysis of the different practices of international humanitarian aid in interaction with national and local practices. In the analysis, we identify the frictions that resulted from the unprecedented situation in Greece. In the discussion section, we interpret our results against the backdrop of the current state of research on practices, structures, and procedures related to humanitarian aid. We argue that in the emergence of frictions, at least three aspects played a crucial role: first, standardized expectations and practices of the international humanitarian aid community; second, the legal framework, which was significantly more complex and politized due to Greece’s EU membership and the concurrent approval of the EU-Turkey Agreement; and third, the ambivalent role of Greece, which on the one hand was affected by austerity policy and massive financial restrictions, especially in its own social and welfare sector, and on the other hand became a recipient country of EU humanitarian aid. We discuss some future developments and further research to be undertaken. In the conclusion, we briefly summarize the paper.

## Financial crisis, austerity, and the role of the Greek government

Neither the refugee crisis nor the deployment of the international humanitarian community occurred in a vacuum. In the following, we will therefore first briefly explain the political context, especially in view of the Greek financial crisis. This will be followed by a presentation of the development of refugee movements in Greece and the reactions of the international community. Before presenting our theoretical framework, we will discuss the ESI in the context of EU humanitarian aid to explain the basis on which the international humanitarian community operated in Greece.

Long-lasting structural deficiencies in the functioning of the Greek state have existed since the 1970s and are widely debated as a cause of the deep impact that the global financial crisis had on Greece from 2007 onwards (Ahamed 2009; Papaconstantinou 2016). The global financial crisis became the European debt crisis and led to the austerity policy of the so-called European Troika of the European Commission (EC), the European Central Bank, and the International Monetary Fund, in 2010. The austerity measures resulted in massive cutbacks, particularly in the social and health sectors but also in other public sectors (Bournakis et al. 2017; Karyotis and Gerodimos 2015; Stuckler and Basu 2014). The consequences for the population were drastic, with the poverty rate rising to the highest in the Euro zone in 2011, an increase in unemployment (Oxfam 2013), bottlenecks in health care, and a rise in the suicide rate (Kondilis et al. 2013).

In Greek society and national policy, Troika’s claims were seen as “neocolonial” (Grahl 2017: 76) and as unacceptable interference in national affairs. In the national elections in 2015, the Greek radical left, the Syriza party under Alexis Tsipras, was able to achieve a surprise victory. Its campaign platform relied on populist arguments about the intended end of austerity (Rori 2016), the restoration of Greek sovereignty, and the representation of the interests of the lower and impoverished middle classes (Stavrakakis 2015). Syriza also took advantage of a campaign conducted by Médecins du Monde as early as 2011, in which the economic crisis and the subsequent austerity policy were framed as a humanitarian crisis, including in the following upcoming negotiations on the second austerity pact (Cabot 2018). Research (Rori 2016) has shown that the popular reputation of Syriza was mainly based on the symbolic effort to try to negotiate new agreements on austerity measures, whereas the real economic and foreign policy successes did not play a major role (Papaconstantinou 2016). Overall, the humanitarian operation under the umbrella of the ESI encountered an extremely tense domestic political situation and can be seen as a perfect occasion for the Greek government to position itself against EU policies.

## Refugee crisis in Greece since 2013

Greece has always been a transit country for refugees due to its geographical location as a gateway to Europe. The number of people arriving usually fluctuates between 50,000 and 60,000 per year. Most people crossed the land border until the construction of the Greek-Turkish border fortifications in 2012, which led to the reform of the Greek asylum system due to pressure from the EC (UNHCR 2014).

As a result of funding shortfalls in refugee aid programs, for instance, of the DG ECHO and the World Food Program (WFP) in the Middle East (Neslen 2014; WFP 2014), as well as the intensification of the war in Syria, the number of refugees in Greece began to rise at the beginning of 2015.

During this time, the central Mediterranean route was the focus of discussion of EU member states because of the massive rise in refugee deaths in boat accidents and broad media coverage. As a result, the European Agenda on Migration was adopted in May 2015 but did not take into account the situation in the eastern Mediterranean and on the Balkan route (European Commission 2015). In June 2015, in view of a renewed funding gap, more than 200 UN organizations and INGOs called on international donors to make announced payments for the Regional Refugee & Resilience Plan in Response to the Syria Crisis (WFP 2015a). At that time, the Balkan route was already heavily frequented, as then-Macedonia and Serbia issued transit visas for refugees. At the end of June 2015, the United Nations High Commissioner for Refugees (UNHCR) issued an appeal to the EU and a Special Mediterranean Initiative (UNHCR 2015), which was intended to draw attention from the central Mediterranean to the eastern Mediterranean and the Balkan route. In July 2015, the feared underfunding of humanitarian aid in the Middle East returned such that the WFP had to announce a further large reduction to its refugee aid efforts in Syria’s neighboring countries (WFP 2015b). The result was a further increase in the number of people seeking protection in Europe. Up to this time, refugees had been looked after and accompanied along the entire Balkan route by volunteers, grassroots organizations, and individual NGOs (Feischmidt et al. 2019). In that period, the UNHCR and INGOs saw responsibility and capacity primarily within the EU and the individual member states rather than regarding this as the actual task for international humanitarian actors.

**Fig. 1:**
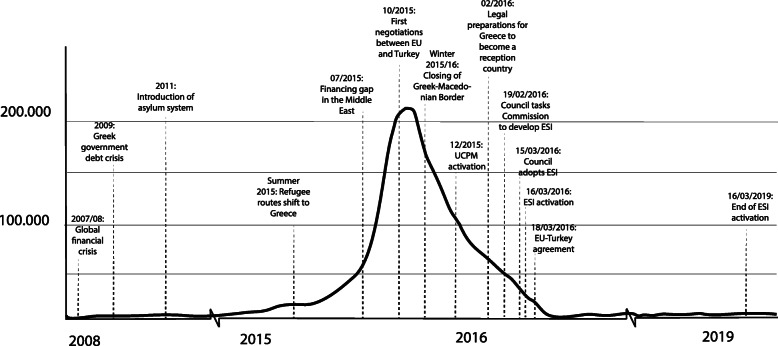
Monthly refugee arrivals to Greece by sea and land, own illustration. Data: UNHCR 2020

At the beginning of September 2015, it became clear that the previous policy of ignoring could no longer be sustained internationally due to the continuing rise in numbers of refugees (see Fig. 1). The situation led to heavy pressure for action at the EU level. UN organizations and INGOs began to become active on a large scale, in some cases for the first time in their history in European territory. In September 2015, the UNHCR published an emergency appeal including an Initial Response Plan for the Refugee Crisis in Europe (UNHCR 2015) and started to operate a transit site in Idomeni at the Greek-Macedonian border in cooperation with INGOs: The International Federation of Red Cross and Red Crescent Societies launched a Disaster Relief Emergency Fund and subsequently an emergency appeal in early September (van Pottelbergh et al. 2019). Not until the end of October 2015 was it decided to support Greece from the EU through funding UNHCR. As it became apparent that the borders in Europe and especially on the Balkan route would be closed, the search began for ways to provide adequate assistance for refugees who were likely to be stranded in Greece. In December 2015, Greece activated the Union Civil Protection Mechanism (UCPM)^1^. However, since the member states themselves had hardly any material and resources at their disposal, this activation was not very successful (European Commission 2018).

At the beginning of 2016, the border with then-Macedonia gradually closed in Idomeni. On 18–19 February 2016, the European Council officially commissioned the EC to develop the ESI, which should “provide humanitarian assistance internally, in cooperation with organizations such as the UNHCR, to support countries facing large numbers of refugees and migrants, building on the experience of the EU Humanitarian Aid and Civil Protection department” (European Council 2016: 5).

On 15 March 2016, the European Council adopted Council Regulation (EU) 2016/369. Only 1 day later, on 16 March 2016, was the ESI activated for the first time for 3 years (2016–2019); 2 days later, the EU-Turkey Agreement was passed (DG ECHO 2018). Thus, the political, financial and institutional basis for transforming Greece from a transit to a recipient country was laid.

## Delivering humanitarian aid in the EU for the first time

In recent decades, the EU has established itself in the international field as one of the central donors of humanitarian aid (Dany 2015; Pariat 2019). In both the Lisbon Treaty and the Consensus on Humanitarian Aid, the EU has committed itself to the humanitarian principles of neutrality and impartiality and a needs-based approach (European Council 2008). However, from its beginnings in the 1990s until the adoption of the ESI, humanitarian aid from the EU was conceived as a policy only for non-EU countries. Operations in member states were not part of the scope of the humanitarian policy at the time (European Council 1996, 2008).

For internal crisis situations, other instruments, such as the UCPM or the Asylum, Migration and Integration Fund, were seen as sufficient. However, these instruments are based on the administrative and operational skills of the member states, cover only certain measures, and are voluntary offers. Until the adoption of the ESI transboundary scenarios that affect the capabilities of member states or internal political and economic crises leading to insufficient response capacities were not covered. Therefore, the ESI opened a new chapter in EU humanitarian aid, making it possible for the first time to carry out long-term EU-financed humanitarian aid operations within the Union.

 The main purpose of the ESI is to supplement the existing voluntary support structures in case of a humanitarian emergency (Miglio 2016) and “to address on a sufficiently predictable and independent basis the humanitarian needs of disaster-stricken people within the Union” (Council of the European Union 2016: 1). The ESI can be activated in the case of a current or potential disaster with an extraordinary range and far-reaching humanitarian consequences in one or many member states and where other instruments are no longer sufficient.

In 2016, the activation of the ESI was followed by rapid implementation. On the day of activation, the first allocations were made within the framework of the ESI and INGOs and UN organizations started their projects (European Commission 2019). The involvement of the DG ECHO, an actor who could draw on existing procedures and long-term working relationships with established partners under the Framework Partnership Agreement (DG ECHO 2015), enabled the rapid implementation of the measures (DG ECHO 2018; European Commission 2018). The main areas of application were the provision and maintenance of camps and the creation of winterized shelters including water, sanitation, and hygiene for over 35,000 people (European Commission 2017c). Other areas of assistance were protection, education, delivery of health services, and the provision of food and non-food items. The ESI was continuously adapted to the current needs on site. Individual measures were handed over to the national authorities or the Directorate-General for Migration and Home Affairs (DG Home) of the EU after some time.

## Humanitarian action as arena and the concept of frictional encounters

To analyze the deployment of actors in international humanitarian aid in new environments, the concept of frictions can be combined with the approach of “humanitarian space as arena” (Hilhorst 2018a; Hilhorst and Jansen 2010) and anthropological and sociological research on the humanitarian “community of practice” (Autesserre 2014; Wenger 2009), which is often referred to as “Aidland” (Mosse 2011).

Humanitarian aid actors can be understood as constituting a community of practice. Communities of practice are mostly “informal and invisible to their participants. They have certain core characteristics. Members share (1) a domain of interest, (2) a community, and (3) common practices” (Autesserre 2014: 47). These include “a shared repertoire of resources: experiences, stories, tools, ways of addressing recurring problems” (Wenger 2009: 1), usually combined with a language of their own (Sampson 2011) and material-spatial practices (Smirl 2015). It is furthermore characterized by a standardized operational culture in which the context, i.e., the local socioeconomic, political and cultural structures, is often disregarded (Dany 2014; Hindmann and Fechter 2011; Schuller 2016; Smirl 2015). Hilhorst (2016, 2018b) describes this effect as “ignorancy,” “creating a legitimate and comforting image of guardian angels coming to the rescue of people in distress” (Hilhorst 2018b: 9).

The humanitarian community of practice can be challenged by different and contradictory expectations, norms, and regulations from the donor as well as from national state actors in the arena of international humanitarian aid where different actors interact. The arena approach can assist in understanding these challenges, as “it focuses on how aid is shaped through social negotiation of actors in and around the aid chain. Within the parameters set by the context and the crisis, these actors, together and in the process of project implementation, shape the everyday realities of humanitarian action” (Hilhorst 2018a: 32). Humanitarian aid actors are embedded in “aid-society relations” (Hilhorst 2016), sociopolitical negotiations, geopolitical considerations, and economic interests whose origins lie primarily in colonial, imperialist, or missionary efforts of the global North in the global South (Roth 2015). At the same time, such actors must be able to act on different and at times contradictory stages if they have to satisfy various demands (e.g., donors, national governments, international organizations, their own organizational agendas) simultaneously (Desportes et al. 2019). These at times conflictive negotiation processes can be observed on the practical level as “frictional encounters” (Björkdahl et al. 2016: 4). The concept of frictions was originally borrowed from military theory (Clausewitz 2006). Frictions are unplanned developments at the operational level that run counter to the actual programmatic ideas, plans, and approaches. Frictions can always occur when different concepts, practices, or actors on different levels meet in one arena. The result of this encounter is unpredictable: “Frictions thus tends to change facts on the ground as it creates new and messy dynamics, agencies, and structures as well as unexpected coalitions” (Björkdahl and Höglund 2013: 295). Frictions draw attention to the manifold interactions of the local and the global—or of the international humanitarian community and the national sovereignty—which simultaneously shape and change the actors involved at all levels. Local and global do not face each other as two poles; rather, they are “representatives of a spectrum of agency that extends from those actors whose sphere of influence and operation is restricted and bound by geography or culture, and those whose sphere of influence and operation extends across boundaries and peoples” (Millar 2016: 32). Of particular relevance to frictions is research that investigates how humanitarian action challenges and redefines existing nation-state structures and their sovereignty in negotiation processes of power (del Valle and Healy 2013; Kahn and Cunningham 2013; Titeca and de Herdt 2011).

Frictions can be observed and analyzed at the levels of actors, discourses, and practices. The actor-centered approach, the analysis of established discourses of international humanitarian aid, and the integration of the practices of national actors make it possible to capture developments analytically and to bring the processual character and agency of all actors involved to the fore. A further advantage of the approach is “that it helps us to understand why and how outcomes differ between settings and to explain why friction can sometimes nullify the impacts of intervention, sometimes produce adverse effects, or even, in some cases, help” (Björkdahl et al. 2016: 204).

## Data collection and method

**Fig. 2:**
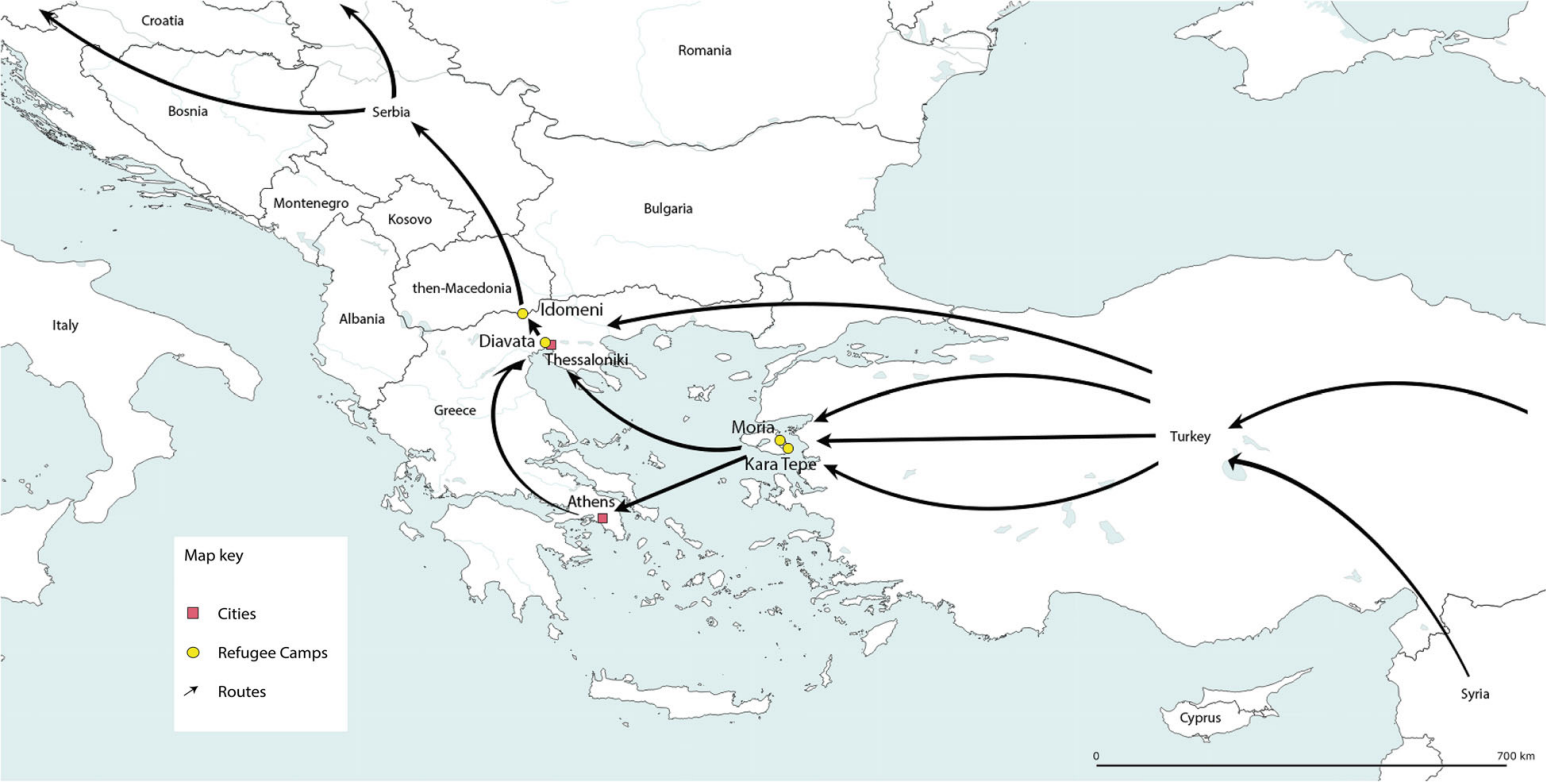
Migratory routes and selected refugee camps. Map: ©Hendrik Schnittker; Geodata: © OpenStreetMap contributors

To answer the research question, we conducted extensive qualitative field research in August/September 2017 and January/February 2019 in Northern Greece (Thessaloniki, Diavata camp, Idomeni), Lesvos (Moria camp, Kara Tepe camp), and Athens (see Fig. 2). In both phases, access to the field was established (1) through existing contacts with the Workers’ Samaritan Federation (ASB), (2) by selection through snowball procedures, or (3) through direct written requests. The ASB had already been active for many years in the Balkans in emergency and refugee aid. With the activation of the ESI for Greece, it opened its own country office and therefore had contacts on the local and national level. Collecting data at two different points in time enabled an adequate depiction of the dynamic situation and different funding phases of the ESI. The first period in 2017 (nine expert interviews) was primarily devoted to exploring the setting and establishing contacts to sharpen the research question and prepare for the extensive data collection. In the second fieldwork phase in 2019, 26 expert interviews were conducted with almost all relevant INGOs in the humanitarian field at the time, as well as with Greek authorities, UN organizations, and other relevant actors in the field. In total, 35 interviews^2^ were conducted. The majority of persons interviewed were country directors or executives at the strategic level (see Table 1).

**Table 1:** Actor category of interviews

Actor category	Interviewees
Greek government officials	1
INGO staff	18
NGO staff	3
UN organization staff	4
DG ECHO staff	2
Grassroot organization staff	5
Other	2

The interviews were based on guidelines, and the topics were structured as follows: description of the organization and current tasks; developments since 2015; experiences, challenges, and assessments of the ESI; comparison of humanitarian operations in Greece with operations in non-European contexts; transferability of Greek experiences to non-European contexts; current developments; and exit strategies. The interviews were recorded and transcribed, or meeting minutes were prepared. Both the transcripts and the minutes were analyzed via qualitative content analysis by using MAXQDA software. The results were contrasted with an extensive literature and document analysis that included scientific publications from research on humanitarian aid, refugee research, and regional studies on Greece as well as reports and policy papers in the public media as well as EU and UN documents. Notably, however, there has been little scientific investigation into this particular case to date. Therefore, the literature has made few references to individual aspects; hence, in part, the analysis was based exclusively on field research and can therefore also be considered a pilot study. Because of the study design with limited time and a gatekeeper position of the ASB reaching only certain actors, the data collection had limitations. It was possible to include most international humanitarian organizations involved in the response, but access to various Greek authorities was difficult to obtain. Therefore, the Greek perspective was supplemented by the INGO narratives and secondary literature. Independent grassroots organizations were only secondarily involved since they were not the focus of the research. Due to lockdown measures after security incidents in the camps as well as administrative restrictions, it was not possible to access camps or interview refugees.

## Analysis: frictions in Greece

The analysis clearly shows that frictions occurred mainly at the operational level of the implementing organizations, which are located directly at the interface between the donor DG ECHO, the national governmental actors, the local conditions in Greece, and the actual needs of the aid recipients.

### Coordination

In almost all interviews, it was stated that one of the main problems of the mission was the coordination between the many different governmental and nongovernmental actors. This was particularly evident in the area of camp management: Due to the primary responsibility of Greek authorities—depending on the camp, different actors such as Greek city administrations, prefectures, the Ministry of Migration Policy, the Ministry of Defense, the Hellenic Army, or even several Greek actors were in charge—only a secondary function as Site Management Support (SMS) officially remained for the UN organizations and the INGOs. However, interviewees (Interview 06 (ASB); Interview 07 (DRC); Interview 14 (ASB); Interview 16 (ASB)) claimed that the Greek authorities were often unable or unwilling to fulfill the camp management role, resulting in massive coordination problems.

These coordination gaps can also be found on other levels: for a long time, it remained highly unclear who was to be responsible for overall coordination. UN humanitarian coordination mechanisms, such as the cluster approach, were not applied in Greece because the Greek government insisted on keeping the coordination in its own hands. At times, different coordination bodies were formed both in the DG ECHO and in the constantly changing institutional structures within the Greek authorities, but these were scarcely able to integrate all efforts into a coherent strategy (Interview 01 (MoMP); Interview 02 (NORCAP); Interview 03 (IOM); Interview 04 (ASB); Interview 05 (UNHCR)).

### Regulations

Regulations were constantly being revised, and responsibilities shifted on both the Greek and EU sides. Decisions made in the DG ECHO headquarters in Brussels also seemed very far away from the operational capacities on the ground; negotiations over money allocations and focus areas were often negotiated “past” the local actors between the DG ECHO headquarters and the headquarters of the INGOs. “It was a large and insurmountable gap” (Interview 07 (DRC)), also between HQs and field offices. The result was an often-criticized audit culture demanded on the part of the EC, which paid close attention to the use of expenditures. In some cases, these very different demands led to “double book systems”: one for the DG ECHO and another for Greek authorities. The fact that the ESI was flanked by a whole range of other nonhumanitarian funds exacerbated this situation and led to a veritable “paranoia of double funding” (Interview 05 (UNHCR)), which made implementation on the operational level considerably more difficult and eventually created gaps in provisions.

### Budgeting

The operation was characterized by short-term budgeting: although it was activated for 3 years from the beginning, the financing was tendered only annually. At the end of each financing phase, it was repeatedly examined whether Greece would be able to assume full responsibility and associated costs. This led to short-term tenders, so personnel had to be dismissed or the organizations had to make advance payments with their own funds on an uncertain legal basis in order to bridge the financing gaps. Wherever this was not possible, especially for smaller INGOs, personnel were unable to extend their contracts, which in turn resulted in costly new job postings and hiring (Interview 08 (ASB)). Here, interviewees (e.g., Interview 07 (DRC)) saw a factor that led to particularly poor cost efficiency for the operation. If the program had been put out to tender for the entire 3 years from the outset, the procurement (e.g., vehicle purchase instead of loan) or the hiring of personnel would have entailed significantly lower costs, the interviewees (e.g., Interview 07 (DRC)) claimed.

### Personnel

The high turnover of INGO personnel due to project-based financing was detrimental to the desired establishment of continuous working relationships and knowledge management, particularly, with local actors. In the initial phase of the operation, knowledge in dealing with DG ECHO structures was particularly necessary. Since only INGOs could be considered DG ECHO framework partners, the personnel were recruited and “flown in” from the pool of international humanitarian aid workers—often of Greek origin but with long-standing expertise in humanitarian aid. The extensive upscaling of personnel resources at some INGOs showed that although they were already involved in Greece before the DG ECHO entered the country, they first dismissed local personnel and then hired them on much worse terms. Therefore, also the international framework partners used local NGOs as implementing partners, which significantly increased the general costs (Interview 01 (MoMP); Interview 07 (DRC)). From the outset, this constellation represented a problem of localization, which became particularly acute through the attempt to successively hand over programs to national actors.

### Capacity building

From the point of view of some INGOs, it also became clear that capacity building was necessary within the Greek government to establish meaningful cooperation and potential program handover (DG ECHO 2018). INGOs such as NORCAP informally had to become “consultants” for the Greek authorities to enable meaningful cooperation (Interview 02 (NORCAP)). In many cases, however, capacity building failed, or the Greek authorities were assumed to have no real interest in capacity building because the respective capacities would have been accompanied by new responsibilities. This resulted in considerable delays in handovers, programs being discontinued, or deterioration in the aid provided (van Pottelbergh et al. 2019).

### Austerity measures and host community support

Due to the austerity measures, however, frictions developed not only from the (supposed) lack of structures on the Greek side but also from the continuation of the austerity measures.

The DG ECHO usually funds activities that include support for host communities (DG ECHO 2019; Ville de Goyet et al. 2012). This was ruled out in Greece due to the specific economic-political situation, as this area is the responsibility of the Greek social and welfare sector affected by the austerity measures of the European Troika and therefore should not be undermined by humanitarian funding of the same commission.

Although strong needs were also evident in the population and were greatly intensified by the financial crisis and the austerity measures (Interview 13 (MdM); Karagkounis 2017), “[t]here were clear instructions from [DG, CD & DFL] ECHO not to support the domestic population with this fund, as would normally have been acceptable in other contexts, as, in theory, this group was being supported by other funds. This had programmatic and operational consequences” (van Pottelbergh et al. 2019: 57). The provisions provided under the ESI were intended to benefit only refugees, asylum seekers, and migrants. As one result, the possible standards for aid, especially in terms of cash assistance and health care for refugees, had to be lowered to national standards to ensure that refugees would not be better off than the local population (DG ECHO 2018; Interview 04 (ASB); Interview 12 (NRC)).

### Standards

Another issue discussed was the implementation of shelter standards. For instance, while Sphere Minimum Standards are supposed to define the level of aid in the global South, it was expected that standards in Greece would have to be raised. In other missions, the minimum standards are often not reached because of constraints by available resources (de Torrenté 2013), whereas in Greece, standards had to be lowered and adjusted to the Greek context (Interview 16 (ASB)). According to interviewee statements, statistics were also “embellished” at times when programs were supposed to be financed only for refugees, but the local population also justifiably made use of them. They were not allowed to appear officially in the statistics due to the lack of a host community support component.

### Restricted support

Since the Greek state is formally responsible for the asylum process and funding via the ESI was conceived as humanitarian aid only, legal aid and specific protection components were not included in the range of services in the first financing phase of the ESI. Required legal support could therefore be provided only to a limited extent or had to be paid from other cost centers (Interview 10 (ARSIS); Interview 11 (OXFAM)). It was not until the second financing phase that the range of services was expanded to include the relevant components (European Commission 2017a).

### Impartiality

The EU-Turkey Agreement led to unequal treatment of different groups of refugees because the agreement covered only refugees who came to Greece from Turkey by sea. While a complete registration system was established on the islands via the “hot spots”, refugees who entered Greece via the Greek-Turkish land border were also to be registered in the so-called Reception and Identification Centre but then to be assisted by the Greek authorities. The actors interviewed assumed that the Greek authorities often failed to register them to avoid any claim to assistance. Without official registration, however, the refugees had no official claim to aid in the camps on the mainland. The consequence of the supply gap was that the INGOs, which did not receive any resources for these refugees, had to be “creative” with the existing resources in order to also be able to supply this group of refugees (Interview 14 (ASB)), but this was often possible only to a very limited extent.

### False context analysis

It was not only the Greek side or political agreements and decisions made responsible for this challenging situation: The analysis shows that there was little or no experience among actors involved in international humanitarian aid in delivering humanitarian aid within the EU: “humanitarians are not used to working with national authorities” (Interview 01 (MoMP)). At least according to critical INGO representatives, the mission in Greece was based on a false context analysis and the false expectation that the “same business as in other places” (Interview 15 (MSF)) would be possible. It became apparent that the “programs didn’t fit the environment” (Interview 09 (UNHCR)) and “failed to adapt to the realities of a European state” (Interview 01 (MoMP)). Being confronted with the actual situation in Greece the question was discussed whether all mission objectives were in line with humanitarian aid principles: “The modernization of the asylum process is not an emergency and therefore not a field of activity for humanitarian aid” (Interview 06 (ASB)).

## Discussion: frictions in the Greek humanitarian arena

With the approach of the humanitarian arena as a place where sociopolitical negotiation processes, geopolitical considerations, economic or party-strategic interests of donors, humanitarian actors, and nation-states meet, it is possible to identify “frictional encounters” (Björkdahl et al. 2016: 4) in the sense of unplanned developments at the operational level that run counter to the actual ideas, plans, and approaches. We have used this approach to identify the frictions as they arose from the deployment of actors involved in humanitarian aid in Greece from 2016 to 2019. The fact that these frictions occurred at all pertains to the structures of the newly formed Greek humanitarian arena and the encounters of the different actors involved.

It is believed that at the beginning of the operation, the Greek authorities assumed that they would be the main recipients of support or at least benefit more from funding (van Pottelbergh et al. 2019). Furthermore, the Greek authorities tried to use the situation to assert or restore their own sovereignty and gain more power, which had been undermined by austerity policies after the financial and debt crises (Alexandrakis 2019). The frictions described here can also be interpreted as an opportunity for the Greek actors to oppose the policies of international humanitarian refugee aid within the bounds of their possibilities, to reinterpret them in their own sense and thus to negotiate the sovereignty that was lost in the financial crisis and the subsequent austerity measures. Over the course of the operation in Greece, a battle was fought in the arena over questions of political sovereignty and the provision of humanitarian aid. The fact that this policy and politics of sovereignty were often purely symbolic—although not without consequences for the practice of humanitarian aid and its moral authority—led to a multitude of frictions for the INGOs that were very resource intensive to negotiate. At least three aspects that contributed to the aforementioned frictions can be identified:

1. The standardized expectations and practices of the international humanitarian aid community
2. The complex legal framework in Greece due to a variety of national and international regulations
3. The ambivalent role of Greece, which on the one hand was affected by massive austerity restrictions and on the other hand was the recipient of humanitarian funds

While the aspects interacted in generating frictions, it is instructive to discuss them separately for analytical clarity and to provide new insights.

### Standardized expectations and practices of the international humanitarian aid community

The understanding of the international humanitarian aid community as a community of practice, also known as “Aidland”, plays a prominent role in contributing to frictional encounters. On the one hand, this situation enabled—as in other operations (Hindmann and Fechter 2011; Schuller 2016)—the “ignorancy” (Hilhorst 2018b: 6) and disregard of the local socioeconomic, political, and cultural Greek context. As a consequence, the mission in Greece was based on the expectation that the operation could be managed with the standardized operational culture consisting of well-known experiences, tools, and resources and that little adaptation was required. On the other hand, the community of practice is also reflected in the way the surveyed actors identified problems with the operation and address local frictions as “recurring problems” (Wenger 2009: 1).

Both the “ignorancy” and the framing of problems can be explained by the lack of coordination and cooperation between different levels and actors that remained a problem for operational actors throughout the Greek mission. The expectations and practices of the international humanitarian aid community and their standardized operational culture were incompatible with the Greek environment and contributed significantly to coordination problems. The lack of coordination, however, is not a peculiarity of the operation in Greece, but a fundamental narrative of the community of practice: “Coordination is always in short supply in Aidland” (Sampson 2011). The struggle of who assumes the lead is an important means to regain state authority that is perceived as threatened by the interference of international humanitarian aid (Kahn and Cunningham 2013). For Greece, this struggle revealed a fundamental conflict between the national government and reservations within the international humanitarian community: The Greek government wanted to take over coordination of the mission and relevant tasks to reestablish sovereignty, but the international humanitarian community questioned whether the Greek side would be able to provide the necessary coordination, personnel, and resources. This situation touches upon the questions of state-society relations and local governance approaches that are critically discussed in humanitarian aid and research (Cunningham 2017; Good et al. 2015; Hilhorst 2018b; Kahn and Cunningham 2013).

The problem of localization—for the Greek case, most obvious in the lack of local partners with expertise and capacity and no transition or exit strategies—has been widely discussed not only in light of the World Humanitarian Summit 2016 and the Grand Bargain but also for decades in the field of humanitarian aid (Glennie and Rabinowitz 2013; Shifting the Power Project 2017; World Humanitarian Summit 2016). One main reason for the failure of localization processes is the standardized operational culture in “Aidland”, in which the inclusion of the context—i.e., the local socioeconomic, political, and cultural structures—is often ignored, even though this is repeatedly demanded in current debates on localization and decolonization (Dany 2014; Hindmann and Fechter 2011; Schuller 2016). Another related problem is who is subsumed under the notion of “local” and thus fulfills the necessary (formal) requirements to continue the established projects in the interests of the donor (Kuipers et al. 2020; Roepstorff 2019; Titeca and de Herdt 2011).

### Complex legal framework in Greece due to a variety of national and international regulations

The frictions due to the responsibility of authorities of an EU member state as well as other legal regulations primarily emerged as conflicting legal and normative standards . “[T]he fact that the operation took place within an EU country, where structures, laws and regulations are in place, challenged humanitarian actors’ standards and approaches” (van Pottelbergh et al. 2019: 56). The clash of different and partly incompatible value and legal systems and regulations—such as regulations of the DG ECHO, other EU laws and regulations, particularly, the EU-Turkey deal, national Greek laws and humanitarian standards and principles such as the Sphere Minimum Standards or the Humanitarian Charter (Sphere Association 2018)—especially posed challenges for the actors. Due to the special situation in Greece, new structures and procedures were negotiated with regard to the role of INGOs, e.g., the role of SMS. These occasionally reflected the legal structure of the Greek constitution rather than humanitarian needs and could not always be filled with concrete corresponding practices at the operational level. The situation revealed a lack of operational agreements with European governments and a lack of experience in dealing with strongly legalized statehood. In contrast, in contexts of limited statehood that usually characterize humanitarian aid (Cunningham 2017; del Valle and Healy 2013), humanitarian aid can be established more easily and without taking other legal regulations into account: “Ironically, the [humanitarian, CD & DFL] sector has been improving its response preparedness tools and instruments over the past years to be able to respond better in contexts where there is a vacuum of political systems. This mentality did not necessarily function in the Greek context” (van Pottelbergh et al. 2019: 56).

### Greece’s ambivalent role between adopting austerity measures and being a recipient of humanitarian aid

For the entire period of the operation, Greece has had a contradictory dual role. While Greece, as a sovereign state, particularly as an EU member state, had the ultimate decision-making powers domestically, the country was by no means able to meet the associated requirements, given the austerity measures that massively affected not only the social and welfare sector but also the administrative structures in state authorities. This was particularly evident in the area of camp management and sectoral coordination. While camp management officially lay in the hands of Greek authorities, INGOs were contracted only to provide SMS. “The Site Management Support (SMS) term was coined to emphasize the leading role of the Greek government in managing the reception sites” (CCCM Cluster 2020: 13). Due to the massive financial cuts in the context of the austerity measures, the Greek government was scarcely capable to carrying out this task, leading to a lack of coordination and decision making. While it is true that the role of SMS is found not only in Greece but also, for instance, in Bangladesh (CCCM Cluster 2020), the Greek case seems to be paradigmatic in the frictions that can arise from this constellation.

The same is true for existing sectoral working groups for INGOs and UN organizations, which face similar problems. “Considering the lack of HCT [Humanitarian Country Team, CD & DFL] or a similar forum, WGs [working groups, CD & DFL] have basically nowhere to suggest guidelines” (CCCM Cluster 2020: 17). Similarly, well-known coordination problems of the humanitarian system as described above were further exacerbated in Greece.

In summary, although the Greek state had de jure decision-making power in all domestic affairs and this could not be undermined due to its distinctive position as a member state of the EU—which also acted as the donor—it was simultaneously subject to a rigid austerity policy due to its obligations to the European Troika. This situation made it impossible to meet these expectations and led to a whole series of frictions, competing interests, and claims of sovereignty in the humanitarian arena.

## Conclusion

This article analyzed the question of what frictions arose in the newly emerged arena of humanitarian aid in Greece, which also marked the first humanitarian operation in the EU. The analysis revealed frictional encounters on different levels. Upon closer analysis, these frictions were revealed to be a nonintentional expression of the negotiation processes of the actors in the Greek humanitarian arena as well as corresponding negotiations about political sovereignty, power, and values. Three aspects emerged as particularly relevant to understanding the case: first, the standardized expectations and practices of international humanitarian aid actors; second, the complex and conflicting legal setting due to a variety of national and international regulations; and third, the ambivalent role of the Greek state as both austerity-ridden and a recipient of EU humanitarian aid.

The ESI was activated for 3 years in 2016. The program was adapted each year and various program parts were transferred to DG HOME before 2019. In March 2019, the last parts of the program were handed over, and the activation of the ESI ended. Questions therefore arise regarding what can be learned from this period and the frictions in Greece and what the future of the instrument and humanitarian action in the EU is.

At present, it is not foreseeable whether the described negotiations of values, power, and positions in the Greek humanitarian arena will have long-term consequences and, if so, what they will be. However, the fear was expressed that the DG ECHO would have lost a part of its humanitarian mandate and thus its independence from other EU institutions with the mission in Greece. The mission in Greece and the ESI as such were controversial among the DG ECHO and INGOs from the outset. Although many actors later supported the mission, it could have led to reputational damage and identity crises among INGOs and the DG ECHO due to the politicization of humanitarian aid associated with the ESI and the close connection with the asylum and migration regime of the EU-Turkey Agreement. Even though the politicization of vulnerability cannot be discussed in this paper, it requires further investigation: vulnerability was given great weight within the framework of the migration and asylum regime resulting from the EU-Turkey Agreement not only as an operational but also as a legal category, and correspondingly, it had vital implications (Freedman 2019; Howden and Kodalak 2018). Although similar instrumentalizations can be found in other contexts (Agier 2016; Lorenz 2018), the entanglement of vulnerability and the asylum process as well as claims for benefits were particularly acute in Greece. This definitely challenges the originally needs-based approach of EU humanitarian aid and the ESI in particular and may contribute to further frictions in the international humanitarian aid community.

The question thus arises as to whether this mission is one of those international humanitarian aid missions that have led to sustained frictions in the field of humanitarian aid itself, such as the mission in Kosovo (Barnett 2013; Rieff 2002).

The significance of the frictional encounters is by no means limited to Greece in the period 2016–2019. Independent of the Greek case, the question arises as to what extent the struggles described in the humanitarian arena and the corresponding frictions in humanitarian aid have also occurred in other contexts of the European refugee and migrant crisis of 2015/2016. For example, previously unknown frictions and negotiations over the relationship between state provision for refugees, civil protection, and disaster control, as well as humanitarian aid, also became apparent in Germany at that time (Dittmer and Lorenz 2019).

The ESI was intended not only for refugees and migrants in Greece but also for generic situations in which “the exceptional scale and impact of the disaster is such that it gives rises to severe wide-ranging humanitarian consequences in one or more Member States and only in exceptional circumstances where no other instrument available to Member States and to the Union is sufficient” (Council of the European Union 2016: 3). One year after the inactivation of the ESI in the refugee crisis, the COVID-19 pandemic led to a newly adapted and modified activation (European Council 2020). In contrast to the initial activation in Greece, the international humanitarian field did not play a major role here. Nevertheless, it is not unreasonable to expect an increase in “atypical crises” (Philipps 2019: 7) in the future, thereby blurring the geographical boundaries of humanitarian aid. Consequently, the question of the use of the “technical expertise and resources of the international humanitarian system” (Philipps 2019: 7) is increasingly prevalent, not only in the EU but also elsewhere in the global North. This would, as others (Bioforce 2020: 23) argue as well, “require [humanitarian, CD & DFL] organisations to reflect on their role and mandate and enhance their ability to work in collaboration with new actors”. With this in mind, the ESI or comparable operations like the one in Greece could first fundamentally shift the balance between humanitarian aid, social services, civil protection, and disaster relief; second, challenge the self-concept of both humanitarian organizations and professionals; and third, result in a fundamentally changed humanitarian arena and new types of frictions elsewhere.

## Data Availability

For privacy and data protection reasons, a differentiated presentation and citation of the individual interviews will be omitted and subsumed under “interviews” listed by different numbers. Due to the very limited number of people working in the contexts described, details regarding the interviewees would always include personally identifiable information. In the appendix, a list of the interviewed organizationsas well as the date and place of each interview can be found. A detailed list of the interviewees is available from the authors upon reasonable request with the interviewees’ consent.

## Appendix

Interviews

Interview 01 (2019) Ministry of Migration Policy (MoMP), Thessaloniki, Greece

Interview 02 (2019) NORCAP, Skype Interview

Interview 03 (2020) International Organisation of Migration (IOM), Athens, Greece

Interview 04 (2019) Workers` Samaritan Federation (ASB), Thessaloniki, Greece

Interview 05 (2019) United Nations High Commissioner for Refugees (UNHCR), Thessaloniki, Greece

Interview 06 (2017) Workers` Samaritan Federation (ASB), Thessaloniki, Greece

Interview 07 (2019) Danish Refugee Council (DRC), Athens, Greece

Interview 08 (2017) Workers' Samaritan Federation (ASB), Foreign Aid Section, Cologne, Germany

Interview 09 (2019) United Nations High Commissioner for Refugees (UNHCR), Athens, Greece

Interview 10 (2017) Association for the Social Support of Youth (ARSIS), Thessaloniki, Greece

Interview 11 (2019) OXFAM, Athens, Greece

Interview 12 (2017) Norwegian Refugee Council (NRC), Thessaloniki, Greece

Interview 13 (2019) Médecins du monde Greece (MdM), Thessaloniki, Greece

Interview 14 (2019) Workers` Samaritan Federation (ASB), Thessaloniki, Greece

Interview 15 (2019) Médecins sans frontieres (MSF), Athens, Greece

Interview 16 (2019) Workers` Samaritan Federation (ASB), Thessaloniki, Greece

Interview 17 (2019) DG ECHO Athens, Greece

Interview 18 (2019) DG HOME, Athens, Greece

Interview 19 (2017) Youth Support Centre, Social Worker, Thessaloniki, Greece

Interview 20 (2017) International Humanitarian Expert, Berlin, Germany

Interview 21 (2017) Workers' Samaritan Federation (ASB), Thessaloniki, Greece

Interview 22 (2017) Women and Health Alliance (WAHA), Thessaloniki, Greece

Interview 23 (2019) Help Refugees, Thessaloniki, Greece

Interview 24 (2019) Workers` Samaritan Federation (ASB), Thessaloniki, Greece

Interview 25 (2019) InterEuropean Human Aid Association (IHA), Thessaloniki, Greece

Interview 26 (2019) Lighthouse Relief, Skala Sikameneas, Greece

Interview 27 (2019) Médecins du Monde (MdM), Mytilene, Greece

Interview 28 (2019) Kara Tepe Camp Management, Mytilene, Greece

Interview 29 (2019) Lesvos Legal Centre, Mytilene, Greece

Interview 30 (2019) International Committee of the Red Cross (ICRC), Mytilene, Greece

Interview 31 (2019) One Happy Family, Mytilene, Greece

Interview 32 (2019) Médecins sans frontieres (MSF), Operational Centre Brussels, Athens, Greece

Interview 33 (2019) German Federal Office for Migration and Refugees, Athens, Greece

Interview 34 (2019) International Rescue Commitee (IRC), Athens, Greece

Interview 35 (2017) Workers' Samaritan Federation (ASB), Foreign Aid Section

## Abbreviations

ASB: Workers’ samaritan federation
DG ECHO: Directorate-general for european civil protection and humanitarian aid operations
DG Home: Directorate-general for migration and home affairs
EC: European commission
EU: European union
ESI: Emergency support instrument
INGO: International nongovernmental organization
NGO: Nongovernmental organizations
SMS: Site management support
UCPM: Union civil protection mechanism
UNHCR: United Nations high commissioner for refugees
WFP: World food program

## Footnotes

1 The objective of the UCPM is to strengthen cooperation between the EU member states and associated partner states, in the field of civil protection and disaster response. In case the scale of an emergency overwhelms the response capabilities of a country, it can request assistance from other states activating the mechanism. Given this architecture, the UCPM requires (a) that other states are willing and able to contribute relief forces or material and (b) that the requesting state has the capabilities to coordinate the incoming assistance (European Commission 2017b).

2 For privacy and data protection reasons, a differentiated presentation and citation of the individual interviews will be omitted and subsumed under “interviews” listed by different numbers. Due to the very limited number of people working in the contexts described, details regarding the interviewees would always include personally identifiable information. In the appendix, a list of the interviewed organizations as well as the date and place of each interview can be found. A detailed list of the interviewees is available from the authors upon reasonable request with the interviewees’ consent.
